# A preliminary study into the effects of the COVID-19 pandemic on Yale-Brown Obsessive Compulsive Scale (Y-BOCS) scores of patients with obsessive compulsive disorder

**DOI:** 10.1192/bjo.2021.668

**Published:** 2021-06-18

**Authors:** Claire Fischer, Hannah Gyekye-Mensah, Ilenia Pampaloni, Augusta Chandler, Anusha Govender, Josephine Sibanda-Mbanga

**Affiliations:** 1 South West London and St George's Mental Health Trust; 2 St George's University of London; 3 South West London and St George's Mental Health NHS Trust

## Abstract

**Aims:**

The COVID-19 pandemic has presented a challenge for treating people with OCD and it could be postulated that those with OCD fearing contamination might be more affected in current circumstances. Although there have been some studies already published, results have been heterogeneous and conflicting; possibly because of different populations or geographical locations examined.

In this preliminary study we aim to identify the impact of the pandemic on the severity of OCD, as measured by Y-BOCS scores. To our knowledge, it is the first UK study of this kind and the only study that examines change in Y-BOCS scores over such a long time period.

**Method:**

Patients were identified from national OCD unit referral databases at Springfield Hospital. Referrals from March 2019–March 2020 were examined and patients included if they had a diagnosis of OCD, were accepted by the service following initial assessment and sufficient data were available. This preliminary study focused only on Y-BOCS to assess clinician-rated severity of OCD. Y-BOCS scores were compared from different time periods correlating to the progression of COVID-19. ‘Pre-pandemic’ score was taken from Jan–Dec 2019 or, if not available, from Jan–23 March 2020 (prior to UK lockdown). ‘Pandemic’ score was taken as the most recent rating from April 2020 onwards.

**Result:**

21 patients were included. All treated as outpatients (although 9 had undergone previous inpatient treatment during the time period above). 81% showed improvement in Y-BOCS score between pre-pandemic and pandemic time periods, with an overall mean decrease in Y-BOCS of 10.3.

**Conclusion:**

Overall, this study indicates that severity of OCD decreased during the pandemic compared to pre-pandemic. It may be that patients found it easier to access remote appointments, or perhaps the pandemic environment of being encouraged to stay at home and limiting unnecessary contact may have allowed limited opportunity for exposure. It might be that the pandemic provided a reason for patients to be avoidant of potential contamination thereby leading to a perceived rather than real improvement in Y-BOCS scores.

Identification of specific contributing factors is beyond the scope of this preliminary study, however it will be important to conduct further research with a larger sample size that incorporates post-lockdown and post-pandemic scores to ascertain whether trends seen here are in fact maintained when normal social contact resumes.

